# Linker 2 of the eukaryotic pre-ribosomal processing factor Mrd1p is an essential interdomain functionally coupled to upstream RNA Binding Domain 2 (RBD2)

**DOI:** 10.1371/journal.pone.0175506

**Published:** 2017-04-07

**Authors:** Fredrik Lackmann, Sergey Belikov, Lars Wieslander

**Affiliations:** Department of Molecular Biosciences, The Wenner-Gren Institute, Stockholm University, Stockholm, Sweden; University of Edinburgh, UNITED KINGDOM

## Abstract

Ribosome synthesis is an essential process in all cells. In *Sacharomyces cerevisiae*, the precursor rRNA, 35S pre-rRNA, is folded and assembled into a 90S pre-ribosomal complex. The 40S ribosomal subunit is processed from the pre-ribosomal complex. This requires concerted action of small nucleolar RNAs, such as U3 snoRNA, and a large number of trans-acting factors. Mrd1p, one of the essential small ribosomal subunit synthesis factors is required for cleavage of the 35S pre-rRNA to generate 18S rRNA of the small ribosomal subunit. Mrd1p is evolutionary conserved in all eukaryotes and in yeast it contains five RNA Binding Domains (RBDs) separated by linker regions. One of these linkers, Linker 2 between RBD2 and RBD3, is conserved in length, predicted to be structured and contains conserved clusters of amino acid residues. In this report, we have analysed Linker 2 mutations and demonstrate that it is essential for Mrd1p function during pre-ribosomal processing. Extensive changes of amino acid residues as well as specific changes of conserved clusters of amino acid residues were found to be incompatible with synthesis of pre-40S ribosomes and cell growth. In addition, gross changes in primary sequence of Linker 2 resulted in Mrd1p instability, leading to degradation of the N-terminal part of the protein. Our data indicates that Linker 2 is functionally coupled to RBD2 and argues for that these domains constitute a functional module in Mrd1p. We conclude that Linker 2 has an essential role for Mrd1p beyond just providing a defined length between RBD2 and RBD3.

## Introduction

Ribosome biogenesis in eukaryotic organisms is evolutionary conserved to a great extent. The outline of the events that produce ribosomes from a large pre-ribosomal RNA (pre-rRNA) molecule has been established in the yeast *Saccharomyces cerevisiae* [1]. The primary transcript of the ribosomal gene, the pre-rRNA, presumably folds in several intermediate states and orderly assembles together with processing factors and ribosomal proteins into of a set of successive pre-ribosomal complexes. While the compositions of the pre-ribosomal complexes have been described [2–7], the functions of the individual components are much less understood.

In *S*. *cerevisiae*, the protein Mrd1p is required to coordinate endonucleolytic cleavage of the pre-rRNA at the three sites: A_0_, A_1_ and A_2_ [8]. U3 snoRNA is also utilized for cleavage at these sites, and Mrd1p is required to liberate U3 snoRNA from the pre-ribosome [9]. Thus, Mrd1p and its homologues in other species [10–15] are essential for synthesis of 40S ribosomal subunits and the formation of 18S rRNA [11–12, 14–16]. It is also required for establishment of nucleoli [16]. Mrd1p homologues have a conserved modular structure consisting of multiple RNA binding domains (RBDs), connected by linker regions [8,9]. While yeast Mrd1p and most homologues in other species have five RBDs, an additional RBD is present in the animal homologue Rbm19 and the homologue in choanoflagellates, inserted between the RBDs corresponding to RBD1 and RBD2 in *S*. *cerevisiae* [10]. RNA-binding proteins often have a modular architecture. Structural studies of how RNA-binding modules recognize and bind their targets have demonstrated the importance of the linker regions to achieve versatility and specificity of binding [17,18]. To understand the function of Mrd1p, it is important to analyse the contribution of each modular domain, i.e. each one of the five RBDs and the four linker regions. Mutational analyses of yeast Mrd1p have demonstrated that individual RBDs are essential (RBD3 and 5) or important (RBD1, 2 and 4) for the function of the protein [9]. A bioinformatic analysis revealed that all the RBDs have specific features that are evolutionary conserved in all eukaryotes and defined consensus sequences for each of the RBDs [10].

In contrast to the RBDs, the importance of the linker regions of Mrd1p have not been investigated previously. The functional contribution of linkers is often overlooked, but linker regions have in many cases been shown to be important for RNA recognition of multi-RBD-containing proteins [18]. *In silico* examination also pointed out evolutionary conserved specific properties for the linker region between RBD2 and RBD3 in Mrd1p, called Linker 2 and corresponding to Linker 3 of Mrd1p homologues containing six RBDs [10]. These properties include a recognizable consensus amino acid sequence and highly conserved residues clustered in several positions; *in silico* predictions also consistently indicate the presence of 4–5 α-helices in Linker 2 in numerous species [10]. These properties are not found in the other linker regions in Mrd1p, which suggests that Linker 2 is unique and may possess specific functions.

In order to test the functional importance of Linker 2, we generated a set of Linker 2 mutants. These mutants demonstrate that Linker 2 is required for 40S ribosomal subunit synthesis and cell viability. Furthermore, certain Linker 2 mutants give rise to instability, resulting in degradation of the full-length protein. Our data support the hypothesis that Linker 2 has a unique role in Mrd1p and underlines the view that Linker 2 cooperates with RBD2 and RBD3 when Mrd1p organizes the pre-rRNA and pre-rRNP into structures that are required for pre-rRNA processing. Our data imply that Linker 2 is functionally coupled to RBD2 and also contributes to contacts with other proteins or with the pre-rRNA.

## Materials and methods

### Yeast strains

The names and genotype of the strains used for this study are presented in S1 Table.

### Cloning and genetic manipulations

Plasmids encoding Linker 2 mutant proteins tagged with C-terminal 6xHis-TEV-2xProt A (HTP), N-terminal 3Hemaglutanin (3HA) or double tagged constructs were made by gap repair [19] of cleaved pAS001 [20] in the strain CAY1045. PCR fragments containing the desired mutations in *MRD1* and homology to the cleaved pAS001 plasmid used for gap repair, were generated by overlap PCR [21]. For Swap 1, yeast genomic DNA was used as a template to generate fragments containing *NOP4* Linker 3. For Swap 2, 5'-ins and 3'-ins, genomic DNA of the insect *Chironomus tentans* DNA was used as a PCR template to obtain fragments containing RBD-1 (the *C*. *tentans* Mrd1p homologue) Linker 1 sequences. Plasmids containing the Scrambled, WNsubst. and AVKsubst. sequences were custom synthesized by Eurofins Scientific. W458A, K480E and K494E were created by site-directed mutagenesis (Quickchange II, Agilent). The FLY001 strain was created by transformation of the strain ASY057 with a *P*_*GAL1*_-3HA-*MRD1-LEU2* PCR fragment containing flanking sequences homologous to the *ADE2* gene locus and selected for *LEU2* prototrophy. FLY002 was created by transformation of FLY001 with a PCR fragment containing the *Kluyveromyces lactis URA3* gene (*klURA3*) flanked by sequences homologous to *MRD1* Linker 2 and selected for URA3 prototrophy. FLY003, FLY004 and FLY009 were created by transformation of FLY002 with PCR fragments containing the Swap 1, Scrambled and 5'-ins mutant sequences flanked by *MRD1* sequences and selected on agar plates containing 0.1% 5-fluoroorotic acid (Duchefa Biochemie). The FLY008 and FLY019 strains were created by transforming FLY003 or Ds1-2b strains with a PCR fragment containing the *klURA3* gene flanked by sequences homologous to the *RPA12* gene. Cells were selected on agar plates lacking uracil at room temperature. The mutants were verified by inability to grow at 37°C. All generated plasmid constructs and mutant yeast strains were verified by sequencing. Expression of tagged proteins from the generated strains or constructs was analysed by Western blotting.

### Growth conditions and yeast transformations

Cells containing the Linker 2 mutant plasmids were grown in synthetic defined (SD) medium containing 2% galactose or 2% glucose. The genomic mutant strains were grown in 1% yeast extract, 2% peptone and 2% galactose (YPG) or 2% glucose (YPD). Yeast cells were transformed using the lithium acetate method according to [22].

### Antibodies

Primary antibodies used for Western blot analysis were: Anti-protein A (Peroxidase anti-peroxidase solube complex, P1291, Sigma), anti-Nop1p/Fibrillarin, MCA-38F3 (EnCor), Anti-HA peroxidase High affinity clone 3F10 (Roche), Anti-C-Myc, 9E10 (Santa Cruz)

### RNA isolation and Northern blot hybridization

Exponentially growing yeast cells were shifted from galactose to glucose containing medium and grown for 8 hours. RNA was isolated from 25 OD_600 nm_ units of exponentially growing yeast cells using the hot phenol method described previously [23]. Roughly 2.5 μg of total RNA from each strain was separated on a 1,5% agarose-formaldehyde gel. Before loading, ethidium bromide was added to each RNA sample at a final concentration of 10 μg/ml. After electrophoretic separation, the RNA was detected using a Gel Doc^™^ Ez Image System (BioRad) and quantified using the Image Gauge V3.3 software. The RNA was subsequently blotted to a nylon membrane (Zeta-Probe GT, Bio-Rad) by capillary transfer and UV cross-linked in a Stratalinker (Stratagene). The oligonucleotide probe (5′ GCTCTCATGCTCTTGCC 3′), specific for the *S*. *cerevisiae* ITS1 between cleavage sites D and A_2_, was end-labeled using γ-^32^P ATP (Perkin Elmer) and T4 Polynucleotide Kinase (Thermo Scientific) and purified using the QIAquick Nucleotide Removal kit (Qiagen). Membranes were hybridized in 0.5M sodium phosphate, pH 7.2, 7% SDS at Tm—9°C, washed and analysed with a Fuji Bio-Imaging analyzer FLA3000 using the Image Gauge V3.3 software.

### Sucrose gradient centrifugations

Exponentially growing yeast cells were shifted from galactose to glucose containing medium and grown for 8 hours. 100 OD_600_ units of exponentially growing yeast cells were incubated with 0.1 mg/ml cycloheximide for 10 minutes at 30°C. The cells were transferred to a 50 ml tube, washed in 5 ml lysis buffer (20 mM HEPES, 10mM KCl, 2.5mM MgCl_2_, 1mM EGTA, 0.1 mg/ml cycloheximide, 1mM DTT, pH 7.5) and frozen in liquid nitrogen. Cells were resuspended in 1 ml lysis buffer, 3 ml of 0.1 mm zirconia/silica beads (BioSpec) were added and cells were lysed by vigorous vortexing for 1 minute, followed by 1 minute incubation on ice, repeated for a total of five times. The lysate was cleared by centrifugation (3,200xg) for 15 minutes and the supernatant was transferred to 1.5 ml centrifugation tubes and further cleared by centrifugation (20,000xg) for 20 minutes. 100 OD_260_ units were layered on a 10–50% sucrose gradient, prepared in lysis buffer without cycloheximide and DTT. Ultracentrifugation was performed in a SW40 rotor (Beckman) for 2h 35 min at 39.500 rpm at 4C. Fractions of 0.5 ml were collected. Of these, 50 μl were used to measure OD_260_ and 250 μl were precipitated by addition of 50 μl 100% trichloroacetic acid and incubation on ice for 20 minutes. The precipitated proteins were washed twice in 400 μl acetone, resuspended in 20μl Laemmli buffer (BioRad) and analysed by Western blot.

### Cycloheximide chase experiment

Cells were grown to an OD_600_ of 0.5. Cycloheximide was added to a concentration of 0.1 mg/ml and samples were taken for extraction of proteins at regular time points. Proteins were analysed by Western blot.

### Isotope free pulse-chase experiment

The pulse-chase experiments were performed essentially as described [24]. Briefly, cells containing an HA-UAG-*mrd1*/Swap 1-FPA or HA-UAG-*MRD1*-FPA plasmid and a plasmid expressing an engineered suppressor tRNA^ome-Tyr^ were grown in raffinose containing medium to OD 600_nm_ 0.5. Galactose was added to 2% concentration to induce transcription of the plasmid. After 20 minutes, O-methyl-tyrosine was added to a final concentration of 1mM to supress the amber stop codon, which induces translation (pulse). After 10 minutes, tetracycline and glucose were added to a final concentration of 350 μg/ml and 2% respectively to prevent further translation (chase). Protein samples were taken at different time points during the pulse-chase experiment. The HA-UAG-FPA and suppressor tRNA^ome-Tyr^ plasmids were a gift from Philip Stelter and Ed Hurt. The *MRD1* or Swap 1 mutant sequences were cloned into the HA-UAG-FPA plasmid by Gap repair [19].

## Results

### Description of the Linker 2 mutants

**Fig 1:**
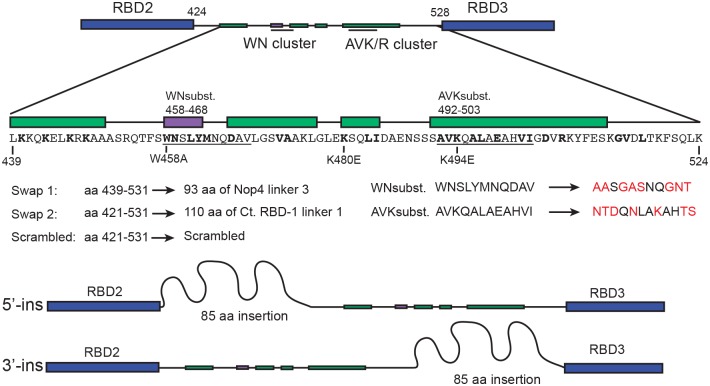
Schematic representation of Linker 2 and the analysed mutations. Linker 2 is positioned between RBD2 and RBD3. Numbers refer to amino acid residue positions in the *S*. *cerevisiae* Mrd1p. Linker definition and consensus sequence as previously described [10]. The most conserved part of Linker 2, between amino acid residues 439 and 524, contains four predicted α-helical regions (green boxes) and one β-strand (purple box). The *S*. *cerevisiae* Linker 2 amino acid sequence shared by homologues in other species is shown in bold. The conserved WN and AVK/R clusters are underlined. Single amino acid residue substitutions (W458A, K480E and K494E) are indicated together with three extensive mutations (Swap 1, Swap 2, Scrambled). In WNsubst and AVKsubst, the substitutions are shown in red and their locations are pointed out above the schematic representation of secondary structure predictions. In two additional mutants, 85 amino acid residues (curved line) were inserted, either between RBD2 and Linker 2 (5′-ins) or between Linker 2 and RBD3 (3′-ins).

Linker 2, located between RBD2 and RBD3 in Mrd1p, is 105 amino acid residues in length and contains several predicted secondary structure elements as well as clusters of highly evolutionary conserved residues within a defined consensus sequence [10] (Fig 1). The functional importance of Linker 2 was analysed by *in vivo* mutational analysis. The mutations are described in Fig 1. In one set of Linker 2 mutants, called Swap 1, Swap 2 and Scrambled, we changed the primary structure, while keeping the length of the linker. In Swap 1, the third linker of Nop4p, another yeast protein containing multiple RNA binding domains, replaced amino acid residues 439–531. In Swap 2, amino acid residues 421–531 were replaced by a part of linker 1 of RBD-1, the *C*. *tentans* homologue of Mrd1p. This sequence is not similar to any region of Mrd1p. In Scrambled, amino acid residues 421–531 were replaced by a segment containing the same amino acid residues, but in a scrambled order. We also changed the amino acid sequence of the evolutionary conserved WN and AVK/R clusters to amino acid residues of different characteristics (WNsubst. and AVKsubst., respectively). We furthermore generated Linker 2 mutants where single conserved amino acid residues were substituted (Fig 1). Namely, tryptophane 458 was substituted to an alanine (W458A). Lysines 480 and 494 were exchanged by glutamic acid residues (K480E and K494E, respectively). To test if the spacing between Linker 2 and the upstream RBD2 or between Linker 2 and the downstream RBD3 is functionally important, we generated insertion mutants. An 85 amino acid residues long segment, originating from linker 1 of the *C*. *tentans* Mrd1p homologue was inserted upstream or downstream of Linker 2 (5'-ins and 3'-ins, respectively) (Fig 1).

The mutant genes were cloned into the plasmid vector pAS001 [20], and transformed into a yeast strain where the wild type (WT) *MRD1* gene is controlled by the *GAL1*-promoter (*P*_*GAL1*_*-MRD1*). Using a second strain, the Swap 1, Swap 2 and 5'-ins mutations were introduced into the genomic *MRD1* gene containing a C-terminal 6xhis-TEV-protein A tag (HTP tag). This strain has an additional genomic copy of WT *MRD1*, controlled by the *GAL1*-promoter (*P*_*GAL1*_*-MRD1*).

### Linker 2 is necessary for cell growth

**Fig 2:**
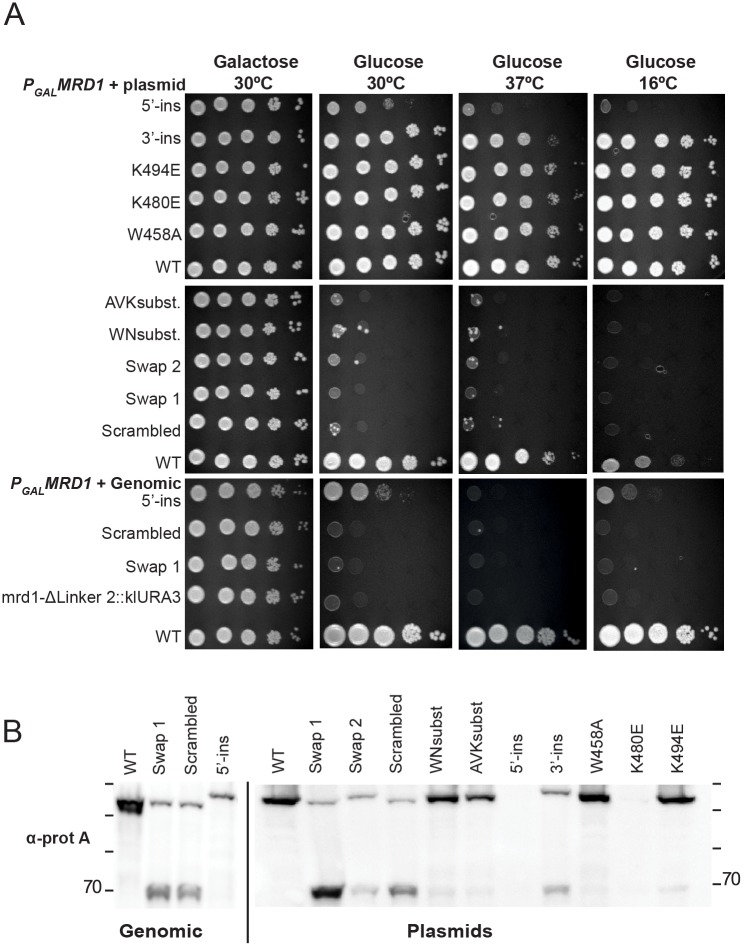
Growth characteristics of Linker 2 mutants and expression of Mrd1 mutant proteins. A. Mutant *MRD1* genes were introduced into yeast cells, either as part of a plasmid (P_GAL_*MRD1*+plasmid) or integrated into the genome (P_GAL_*MRD1*+Genomic). In both cases, a WT *MRD1* gene was present in the genome, controlled by a *GAL1* promoter. The cells were diluted in steps (10 times for each step) and pipetted onto agar plates (from left to right), containing either galactose or glucose, followed by incubation at 30°C, 37°C or 16°C. Mutants are indicated to the left of each dilution series. The parental strain *mrd1-ΔL2*::*klURA3*, (FLY002, See S1 Table), bearing a non-functional *MRD1* gene, served as a negative control for cell growth. B. Western blot analyses of the expression of Mrd1p in WT and mutant strains. Extracts from approximately 5x10^5^ cells were used. Ponceau staining demonstrated that approximately equal amounts of total proteins was loaded in each well (data not shown). The WT or mutant *MRD1* genes were either present in the genome or in a plasmid. The protein A part of the HTP tagged proteins was used for detection. WT Mrd1p is 101 kDa and its HTP tag is approximately 17 kDa. Migration of size marker proteins are shown (180, 130, 100, 70, kDa).

Growth of the Linker 2 mutants was tested on agar plates containing glucose at 30°C, 37°C and 16°C (Fig 2A). The WNsubst, AVKsubst, Swap 1, Swap 2 and Scrambled mutant strains showed a lethal phenotype at the three temperatures. 5’-ins had a slow growth phenotype at 30°C that was more severe at 16°C and lethal at 37°C. This was more clearly seen after longer growth periods (data not shown) and for the genomic 5'-ins as compared to the plasmid encoded 5'-ins (Fig 2A). 3’-ins had a mild growth defect at all three temperatures, although hardly detectable when grown on plates for longer periods (Data not shown and Fig 2A, 37°C). None of the single amino acid residue substitutions (W458A, K480E, K494E) had any observed effect on cell growth at any temperature tested. The mutants expressing plasmid encoded Linker 2 mutant proteins showed similar growth defects as their genomically expressed counterparts. Western blotting demonstrated that all the mutants strains expressed the full-length Mrd1p variants, but at different levels (Fig 2B). For the K480E mutant and the plasmid encoded 5′-ins, the mutant proteins were present in very small amounts, although detected after longer exposures (data not shown). There was no correlation between expression levels and growth phenotypes (see Discussion). We also observed that in several of the mutant strains, expression of the mutant gene, in addition to full-length Mrd1p, also resulted in accumulation of an approximately 70 kDa fragment. This was particularly pronounced in Swap 1, Swap 2, Scrambled and 3’-ins (Fig 2B). We refer to this polypeptide as “70 kDa fragment”. The levels of the 70 kDa fragment relative to full-length protein varied between experiments, but it was typically the predominant form of the protein for the Swap 1 and Scrambled mutants, whereas its levels were lower in Swap 2 and 3'-ins mutants. The size of the truncated form was similar in the different mutants.

From the growth phenotypes, we conclude that Linker 2 is essential. All gross changes (Swap 1, Swap 2 and Scrambled) of the primary sequence were incompatible with cell growth. The same was true for WN and AVK/R cluster mutations, whereas individual amino acid residue substitutions had no effect on cell growth (See Fig 2A). This argues for that combinations of amino acid residues in Linker 2, at least within the WN and AVK/R-clusters, are essential. We further conclude that the spatial relation of Linker 2 relative to RBD2 and RBD3 is important, especially so for RBD2 (see Discussion). Interestingly, 5’-ins had a more severe growth phenotype than an Mrd1p mutant lacking RBD2 (ΔRBD2), most notably at 30°C and 37°C (S1A Fig). Furthermore, 5'-ins was unable to support growth at 37°C on galactose containing plates, which allows co-expression of wild type Mrd1p (S1B Fig). This suggests that 5'-ins has a dominant negative phenotype when grown at 37°C.

*P*_*GAL1*_*-MRD1* cells that stop growing after depletion of Mrd1p in glucose containing medium can start growing upon resumed expression of Mrd1p in the presence of galactose. We tested if the presence of the genomically expressed Linker 2 mutant proteins influenced the recovery of *P*_*GAL1*_*-MRD1* cells when shifted from glucose to galactose containing medium (S1C Fig). Scrambled and Swap 1 mutants slightly enhanced the recovery at 16°C and 37°C and were comparable to the WT control, suggesting that these proteins may be partly functional. In contrast, 5'-ins did not enhance the recovery at 16°C and 30°C. At 37°C, the recovery was inhibited in the presence of 5'-ins, again suggesting that 5'-ins has a dominant negative phenotype at 37°C.

### The growth defects of linker 2 mutants correlated with pre-rRNA processing defects

**Fig 3:**
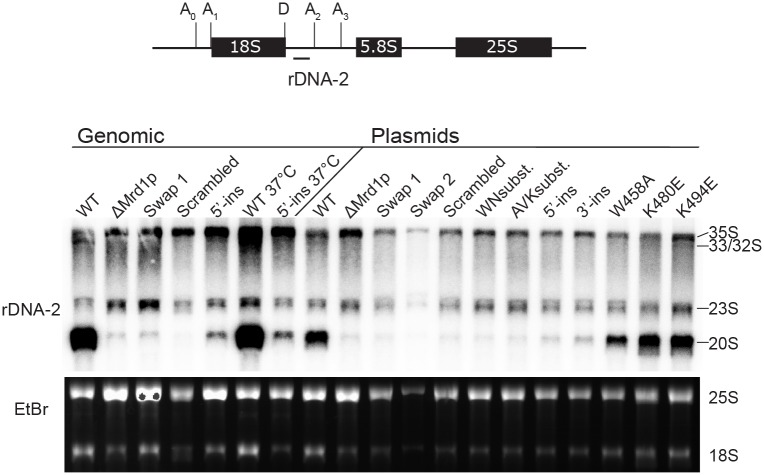
Northern blot analyses of total RNA from Linker 2 mutants. Total RNA was extracted from each Linker 2 mutant as well as from cells bearing a WT *MRD1* gene and separated in an agarose gel under denaturing conditions. The gel was stained with ethidium bromide (shown at the bottom of the figure) to visualize and quantify 18S and 25S mature rRNA. The RNA was transferred to a nylon membrane and hybridized with a ^32^P-labeled oligonucleotide probe complementary to a sequence present between cleavage sites D and A_2_ in 35S pre-rRNA (Schematically shown on top). The positions of 35S, 33/32S, 23S and 20S pre-rRNA are indicated to the right. WT or mutant *MRD1* genes present either in the genome (Genomic) or on a plasmid (Plasmids) are indicated above each lane. All cells were grown at 30°C prior to RNA extraction, unless otherwise indicated (WT 37°C and 5'-ins 37°C).

Mrd1p is essential for cleavages at sites A_0_, A_1_ and A_2_ that generate the 20S and 27SA_2_ pre-rRNAs and, thus, releasing pre-40S and pre-60S ribosomal subunits [8]. We therefore tested if the growth defects of the Linker 2 mutants correlated with the amounts of 18S rRNA and with pre-rRNA processing defects at sites A_0_, A_1_ and A_2_. Ethidium bromide staining of 25S and 18S mature rRNA showed reduced levels of 18S rRNA relative to 25S rRNA in all Linker 2 mutants (Fig 3 and S2A and S2D Fig), except in the single amino acid residue substitution mutants. Northern hybridization was performed using a probe positioned between site A_2_ and D in the pre-rRNA (Fig 3). As compared to the respective WT controls, the 20S pre-rRNA levels were drastically reduced in all Linker 2 mutants that displayed growth defects and reduction of 18S (S2B Fig). Single amino acid residue substitution mutants, K480E, K494E and W458A, which did not show any growth defects, had 20S pre-rRNA levels similar to those of WT cells. (S2B Fig). 35S pre-rRNA was accumulated in the genomic mutants as compared to WT cells (Fig 3) and with the exception of the single amino acid residue substitutions, 35S pre-rRNA was also slightly accumulated in the plasmid expressed mutants in relation to WT cells (Fig 3 and S2C Fig). Furthermore, no 33/32S pre-rRNA was detected in the genomic mutants nor in most plasmid mutants. Low levels of 33/32S pre-rRNA species were detected in the WT control expressed from a plasmid (Fig 3). Similar low levels of 33/32S pre-rRNA were detected in K480E, K494E and W458A, but none were detected in the other mutants. Collectively, the analyses of pre-rRNAs and mature rRNAs show that Linker 2 is required for pre-rRNA processing at sites A_0_, A_1_ and A_2_.

### Linker 2 mutant Mrd1 proteins are incorporated into pre-ribosomes

**Fig 4:**
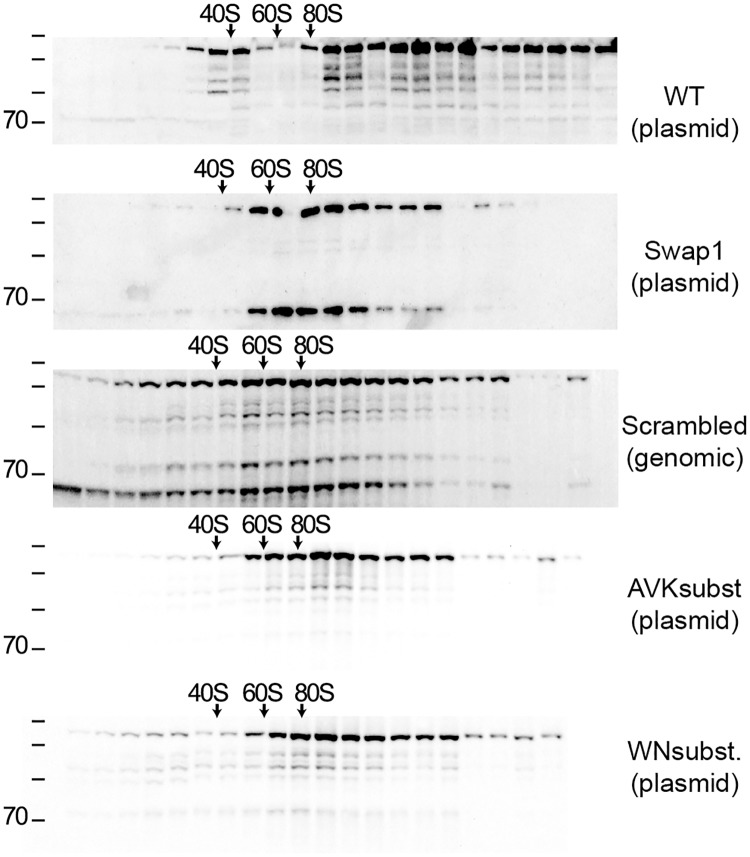
Sucrose gradient centrifugation analyses of Linker 2 mutants. Extracts from cells expressing WT Mrd1p or mutated Mrd1 proteins were centrifuged in a 10–50% a sucrose gradient and fractions were collected. Absorbance at 260 nm and positions of 25S and 18S rRNAs determined by Northern analyses were used to locate 80S ribosomes and 60S and 40S ribosomal subunits. Proteins in each fraction were analysed by Western blot. The tagged Mrd1 proteins were detected with anti-protein A antibody. Migration of size marker proteins is shown to the left (180, 130, 100, 70, kDa).

The lethal Linker 2 mutants had a similar phenotype as complete absence of Mrd1p. We therefore analysed the distribution of Swap 1, Scrambled, WNsubst. and AVKsubst. mutant Mrd1 proteins in sucrose gradients following ultracentrifugation (Fig 4) and found that they sediment together with 80-90S complexes. This argues that these Linker 2 mutations did not prevent the incorporation of Mrd1p into pre-ribosomal processing complexes, but rather that in these complexes they are unable to perform the normal function of Mrd1p. We noted that the distribution of Mrd1p was somewhat influenced by mutations in Linker 2. As compared to WT Mrd1p, the Linker 2 mutant proteins were present to less extent in complexes larger than 80S, and some Linker 2 mutant proteins, for example Scrambled, were present to greater extent in complexes smaller than 40S. These changes in distribution presumably reflected influence on assembly and/or processing of pre-ribosomes. Similar effects have been observed in RBD mutants [9]. The 70 kDa fragment was found co-sedimenting with the full-length protein and 80-90S complexes in Swap 1 and Scrambled mutants. We could also detect a small amount of the 70 kDa fragment at the top of the gradient, especially in Scrambled (Fig 4).

### Linker 2 is important for the stability of Mrd1p

**Fig 5:**
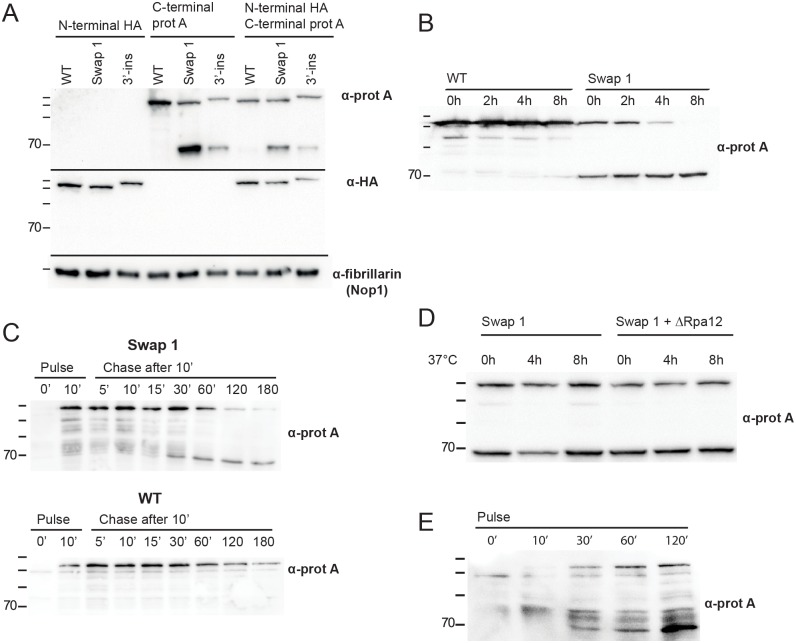
**A. Western blot analysis of *MRD1* mutant constructs.** Protein extracts from strains expressing WT and mutant Mrd1 proteins tagged with either an N-terminal HA-tag or a C-terminal HTP tag or with both tags were subjected to Western blot analyses of two parallel gels. After blotting, one membrane was probed with anti-protein A antibody and the other with anti-HA antibody. As a loading control, one of the membranes was stained with an anti fibrillarin antibody. Migration of size marker proteins is shown to the left (180, 130, 100, 70, kDa). **B. Stability of Mrd1p containing the Swap 1 mutation.** Cells expressing either WT Mrd1p or Swap 1 mutant proteins were treated with cycloheximide starting at 0h. Protein samples were taken at the indicated time points and analysed by Western blot. Migration of size marker proteins is shown to the left (180, 130, 100, 70, kDa). **C. Pulse-chase analyses of Mrd1 Swap 1 mutant proteins.** Synthesis of HA-UAG-Mrd1/Swap 1 protein was achieved by induction of transcription and translation by addition of galactose and by suppression of an amber stop codon, respectively. Translation was inhibited and protein samples were taken at the indicated time points and analysed by Western blotting. Anti-protein A antibodies detected HA-O-methyl-Tyr-Mrd1/Swap 1-FlagProteinA (Top) and HA-O-methyl-Tyr-Mrd1-FlagProteinA (Below). Lanes 1–2 show the time points before and after induction of translation for 10 minutes. Lanes 3–9 show the chase time points, starting after the 10 minutes pulse. Migration of size marker proteins is shown to the left (180, 130, 100, 70, kDa). **D-E. Formation of the 70 kDa fragment in the absence of RNA pol I transcription. D.** Swap 1 mutant cells and Swap 1 mutant cells in Δrpa12 background were shifted from 30°C to 37°C at time point 0h. Protein samples were extracted after 4 and 8 hours and analysed by Western blot. **E.** Pulse experiment of the Swap 1 mutant protein in Δrpa12 background at 37°C. Δrpa12 cells, containing the HA-UAG-*mrd1*/Swap 1-FlagProteinA construct, were shifted from 30°C to 37°C and incubated for 120' minutes. The cells were subsequently pulsed, starting at time point 0’. Proteins were extracted at the indicated time points and analysed by Western blotting, using anti-HA antibody. Migration of size marker proteins is shown to the left (180, 130, 100, 70, kDa).

Based on the results in Fig 2B, indicating the accumulation of a 70 kDa fragment in the mutant cells, we hypothesized that the mutant proteins were unstable. We therefore performed Western blot analyses of the lysates from the strains bearing plasmid encoded Swap 1 and 3'-ins proteins containing C-terminal HTP-tags, N-terminal HA-tags or both N-terminal HA and C-terminal HTP-tags. With an anti-HA antibody we could only detect the full-length protein of both single and double-tagged constructs and, thus conclude that no stable fragment containing the N-terminal part of the protein was formed (Fig 5A). In contrast, with an anti-protein A antibody the 70 kDa fragment was readily detected in both single and double-tagged constructs (Fig 5A). A myc-tagged variant of Swap 1 also predominantly revealed a 70 kDa fragment (data not shown) demonstrating that the generation of the 70 kDa fragment, is not due to the HTP-tag at the C-terminus. Thus, collectively our data argues that the 70 kDa fragment observed in the mutant strains contained the C-terminal tag and therefore represents a protein truncated from the N-terminus.

We further characterized the Swap 1 mutant protein as a representative of the Linker 2 mutant proteins accumulating the 70 kDa fragment. The stability of the Swap 1 mutant protein was analysed by Western blot in protein extracts at different time points after translation was shut off in cycloheximide treated cells (Fig 5B). The mutant full-length protein was unstable compared to wild type Mrd1p over a time interval of 8h. The amount of 70 kDa fragment on the other hand appeared to increase by time indicating that it was formed upon degradation of the full-length protein. This was verified by using an isotope-free pulse chase system [24]. Protein samples were taken at different time points and analysed by Western blot (Fig 5C). Prior to addition of O-methyl tyrosine, no Swap 1 mutant protein is synthesized. After 10 minutes of pulsing a band corresponding to the full-length Swap 1 mutant protein was detected, but no 70 kDa fragment was seen. After a 30-minute chase the 70 kDa fragment appeared. The ratio of the 70 kDa fragment to full-length Swap 1 mutant protein increased at later time points. As the 70 kDa fragment appeared after translation had been shut off, we conclude that it is derived from the full-length protein.

As the 70 kDa fragment was present in 90S complexes (Fig 4) we hypothesized that it the full-length Mrd1p was subject to degradation in a pre-ribosomal complex. In order to halt rRNA transcription we deleted the *RPA12* gene in a Swap 1 background. *RPA12* encodes a Pol I subunit that is non-essential for rRNA transcription at 25°C but essential at 37°C. We reasoned that if specific degradation of mutant Mrd1p resulting in formation of the 70 kDa fragment took place in a pre-ribosomal complex, the full-length Swap 1 mutant would be stabilized upon shift to 37°C. No such stabilization was seen however (Fig 5D). Furthermore, we deleted *RPA12* in the strain used for isotope-free pulse chase experiments and performed a pulse experiment after growing cells at 37°C for 2 hours (Fig 5E). The 70 kDa fragment was still formed under these conditions, suggesting that it can be formed in the absence of pol I transcription.

## Discussion

### Linker 2 is essential for Mrd1p function

Mrd1p is an essential ribosomal processing factor [8] that interacts with the pre-rRNA close to the small subunit pseudoknot [20] and is required for release of U3 snoRNA from base pairing within the pre-ribosome [9]. Mrd1p contains five RBDs that all contribute to the overall function of the protein, but the functions of the four linker regions are not known. In recognition of RNA by multi-RBD proteins, the linker regions often play an important role [18]. We have characterized a series of mutations in Linker 2 in yeast Mrd1p *in vivo* and demonstrated that this domain is essential for the overall function of Mrd1p. This is in agreement with the previous *in silico* observations that Linker 2 has evolutionary conserved features such as length of a consensus sequence with clusters of highly conserved amino acid residues and secondary structure prediction similarities [10]. Guided by evolutionary conservation, we introduced mutations in two clusters of conserved amino acid residues, eleven and twelve residues long and also individually substituted three highly conserved residues (See Fig 1). The two clusters of conserved amino acid residues within the linker were found to be required for full function of Mrd1p while the individual residues were not essential in the analyses employed. Several of the Linker 2 mutant proteins, Swap 1, Swap 2, Scrambled, 5′-ins, 3′-ins and K480E, were expressed at lower levels than WT Mrd1p (Fig 2B). The lower expression levels could partially explain the growth phenotypes of the Linker 2 mutants. However, the K480E mutant protein that was expressed at a low level, did not affect cell growth (Fig 2A) or lead to defects in pre-rRNA processing (Fig 3). Notably, the WNsubst. and AVK subst. mutant proteins were expressed at levels comparable to WT Mrd1p, but did not support cell growth or pre-rRNA processing (Figs 2A, 2B and 3). Extensive changes of the amino acid sequence of Linker 2 reduced cleavage at pre-rRNA processing sites A_0_, A_1_ and A_2_, resulting in impaired synthesis of 18S rRNA and loss of cell viability (See Figs 2 and 3). Sucrose gradient experiments showed that the non-functional Linker 2 mutant proteins were integrated in 80-90S ribosomal complexes, which indicates that the mutations compromised a specific Mrd1p function in pre-ribosomal processing rather than its ability to be incorporated in the pre-ribosomes (Fig 4). Furthermore, certain Linker 2 mutants give rise to mutant protein instability, resulting in degradation of the full-length protein (Fig 5A–5E). We conclude that Linker 2 contributes to the essential function of Mrd1p in pre-ribosomal processing.

### Functional coupling of Linker 2 to the neighbouring RBD2

Two mutants analysed in this study, 5'-ins and 3'-ins, were designed to disrupt the potential collaboration of Linker 2 and its upstream and/or downstream RBD. The 3′-ins mutant reduced 20S pre-rRNA levels and affected Mrd1p stability, although cell growth was only mildly affected (Figs 3 and 5A). The 5′-ins mutation had more pronounced functional consequences. It severely reduced 20S pre-rRNA levels and drastically impaired cell growth at all temperatures tested, especially at 37°C where this mutation is lethal. Furthermore, the 5′-ins mutant was dominant negative at 37°C, which was not the case for the other mutants analysed in this study (See Figs 2A and 3 and S1 Fig).

The fact that an insertion between Linker 2 and RBD2 has severe consequences (Figs 2A and 3), whereas an insertion between Linker 2 and RBD3 is largely tolerated, suggests that RBD2 and Linker 2 cooperate during pre-ribosomal processing. It has been reported that structured linker regions of RBD containing proteins can aid in RNA binding by direct participation in RNA binding or by stabilizing adjacent RBDs [18]. Secondary structure predictions suggest that linker 2 is structured (Fig 1) and it is therefore possible that RBD2 and Linker 2 are folded into a structural and functional module within Mrd1p. It is also possible that upon interaction of RBD2 with RNA, Linker 2 becomes further structured and extends the RNA binding platform, as such examples have been reported [18]. RBD2 is required for optimal cross-linking of Mrd1p to helix 27 in the 18S portion of the pre-rRNA [20]. The weak cross-linking to helix 27 in the absence of RBD2 [20], could be dependent on Linker 2-RNA interactions and possibly attributed to clusters of conserved amino acid residues such as the essential WN and AVK/R clusters or the lysine-rich stretch in the immediate proximity to RBD2 (Fig 1).

Interestingly, 5'-ins had a more severe growth defect than an Mrd1 mutant lacking RBD2 (ΔRBD2) (S1A Fig). It is therefore likely that 5'-ins compromises the function of Linker 2 as well as the coordinated activity of RBD2 and Linker 2. The dominant negative phenotype of 5'-ins is indicative of a gain of function of the 5'-ins mutant protein at 37°C. Mrd1p, Nop7p and Nsr1p have been suggested to participate in the formation of the central pseudoknot and to dissociate from the pre-rRNA prior to termination of the full-length pre-rRNA [20, 25,26]. Increased binding to rRNA resulting in inability to dissociate and/or steric hindrance due to insertion of 85 amino acid residues in the vicinity to the Nop7p binding site could explain the dominant negative phenotype of 5'-ins at 37°C.

RBD2 has previously been shown to be important and RBD3 to be essential for Mrd1p function [18]. These domains need to be present together in the same protein for proper Mrd1p function, which is in contrast to, for example, RBD1 and RBD2 that can support Mrd1p function even when present in two separate proteins [9]. Since 3'-ins only mildly impair cell growth, we conclude that although RBD2 and RBD3 need to be present in the same protein they can be separated by more than 200 amino acid residues. This suggests that RBD2 and RBD3 interact with each other, as in such a case a separation in primary sequence may not result in spatial separation [27].

In a number of proteins containing multiple RBDs, neighboring RBDs and their separating linker are known to cooperatively bind RNA to achieve high specificity and affinity, which is required since the binding of individual RBDs to RNA is rather weak and non-specific [28]. In the presence of a short linker, binding of an initial RBD can tether an adjacent RBD into close proximity to its target, resulting in cooperative binding. Linker 2, however, is rather long and an even further increase of length of Linker 2 is tolerated (3'-ins). Furthermore, RBD3 lacks aromatic amino acid residues at critical positions in the RNP1 and RNP2 motifs involved in canonical RBD binding to RNA [10], which suggests that it may not be involved in RNA binding. It is therefore possible that RBD3 is interacting with proteins rather than RNA, which could also be the case for Linker 2 that contains essential clusters of conserved amino acid residues. Therefore, an attractive model is that the RBD2-Linker 2-RBD3 arrangement could be an example where RBD domain variants and a connecting interdomain have co-evolved to contribute to coordinated RNA and protein interactions in a pre-ribosomal RNA-protein complex.

### Linker 2 is required for stability of Mrd1p

The presence of an C-terminal 70 kDa fragment and the reduced levels of full-length protein in several of the Linker 2 mutant proteins suggest that mutation of Linker 2 induces protein instability. As investigated for the Swap 1 and 3′-ins mutants, the N-terminal part of the mutant protein was degraded, leaving a C-terminal 70 kDa fragment that is approximately as stable as WT full-length Mrd1p (Fig 5A and 5B). The degradation presumably does not take place in a pre-ribosomal complex as the 70 kDa fragment can be generated in the absence of rRNA transcription (Fig 5D and 5E). Notably, the mobility of the 70kDa fragment was not visibly different in the mutants. This indicates that in all mutants, degradation of the N-terminal part took place up to approximately the same position in the protein. As the mutant 3’-ins has 85 amino acid residues inserted downstream of Linker 2 and still produced the same size fragment as the other mutants, the 70 kDa fragment is likely to span a region starting just downstream of Linker 2 and extending to the C-terminus. In the pulse-chase experiments, multiple fragments with sizes between full-length protein and the 70 kDa fragment were detected at short chase time points (Fig 5C). These short-lived fragments presumably represent degradation intermediates.

The 70 kDa fragment co-sedimented with pre-ribosomal complexes in sucrose gradients at steady state. Indirectly, this suggests that an Mrd1 protein lacking RBD1, Linker 1, RBD2, Linker 2 and possibly part of RBD3 contains elements sufficient for the protein to associate with pre-ribosomal complexes. The N-terminal part of Mrd1p, containing RBD1-3, has previously been shown to be sufficient for incorporation into pre-ribosomal complexes as well as direct binding to the pre-rRNA [20]. Furthermore, Mrd1p mutants lacking individual RBDs are all incorporated into pre-ribosomal complexes [9]. We conclude that Mrd1p contains multiple domains that allow incorporation into pre-ribosomal complexes.

It is plausible that major changes of the local structure of Mrd1p caused the Mrd1p instability. This may be achieved either by gross changes in Linker 2 (Swap 1, Swap 2 and Scrambled) or by inserting 85 amino acid residues downstream of Linker 2 (3'-ins). Inserting the same sequence upstream of Linker 2 (5'-ins) is apparently tolerated with regard to protein stability. We observed that trace amounts of the 70 kDa fragment was detected also in the more subtle mutants: WNsubst., AVKsubst., W458A, K494E (Fig 2B). Notably, no similar degradation event is observed in *MRD1* mutants lacking the individual RBDs [9]. Thus, our data suggest a specific role of Linker 2 in maintaining stability of Mrd1p.

The protein instability resulting in generation of the 70 kDa fragment may have functional consequences for the Mrd1 protein. However, 3'-ins, which generated moderate levels of the 70 kDa fragment only displayed a mild growth defect, while 5'-ins, WNsubst. and AVKsubst. mutations caused severe growth defects, yet did not result in protein instability. We thus conclude that in addition to an essential function within the pre-ribosome, which is dependent on the WN and AVK/R clusters, Linker 2 is involved in maintaining Mrd1p stability.

## Supporting information

S1 Fig **(A) Growth characteristics of the ΔRBD2 and the 5**′**-ins mutants.** WT cells and cells having mutant Mrd1 proteins, either lacking RBD2 (ΔRBD2) or having an insertion upstream of Linker 2 (5′-ins), were grown over night in glucose containing medium at 30°C and dilutions series (10 times dilution at each step) were pipetted onto agar plates containing glucose followed by incubation at the indicated temperature. **(B) Growth characteristics of cells expressing both the WT Mrd1 and the 5**′**-ins mutant Mrd1 proteins.** Cells containing either a *P*_*GAL1*_ regulated WT *MRD1* gene and a WT *MRD1* gene or a *P*_*GAL1*_ regulated WT *MRD1* and a 5′-ins mutant *MRD1* gene, were grown in galactose containing medium and pipetted onto agar plates containing galactose followed by incubation at the indicated temperature. **(C) Recovery of cell growth after Mrd1 depletion. Linker 2 mutant strains and a *P***_***GAL1***_***-MRD1* strain, were depleted of WT Mrd1 by overnight growth in glucose containing medium.** Synthesis of WT Mrd1 was resumed by pipetting dilution series (10 times dilution at each step) onto agar plates containing galactose followed by incubation at the indicated temperature. The wild type strain PLY094 served as a control for cell growth.
(PDF)

S2 Fig **(A) Reduced levels of 18S rRNA in the Linker 2 mutants that display growth defects.** Quantification of the gel from Fig 3. **(B) 20S pre-rRNA levels are drastically reduced in all Linker 2 mutants that display growth defects.** Quantification of the gel from Fig 3. **(C) 35S pre-rRNA is accumulated in the mutants that display growth defects.** Quantification of the gel from Fig 3. **(D) Additional quantification of 18S versus 25S for the strains with genomic *MRD1* mutant genes.** Since the EtBr image in Fig 3, generated by Gel Doc^™^ Ez Image System (BioRad) contained overexposed pixels and also to verify the method to measure band intensity used, we ran another quantification experiment, which showed essentially the same results. Columns show quantification of 18S/25S ratios with error bars showing the average deviation of double samples.
(PDF)

S1 Table Names and genotypes of strains used in this study. (PDF)
