# Supplementary material for: Linker 2 of the eukaryotic pre-ribosomal processing factor Mrd1p is an essential interdomain functionally coupled to upstream RNA Binding Domain 2 (RBD2)
Source: PLoS One. 2017 Apr 7;12(4):e0175506. doi: 10.1371/journal.pone.0175506 (PMC5384785; doi:10.1371/journal.pone.0175506)
Supplement: S1 Fig — (A) Growth characteristics of the ΔRBD2 and the 5′-ins mutants. WT cells and cells having mutant Mrd1 proteins, either lacking RBD2 (ΔRBD2) or having an insertion upstream of Linker 2 (5′-ins), were grown over night in glucose containing medium at 30°C and dilutions series (10 times dilution at each step) were pipetted onto agar plates containing glucose followed by incubation at the indicated temperature. (B) Growth characteristics of cells expressing both the WT Mrd1 and the 5′-ins mutant Mrd1 proteins. Cells containing either a PGAL1 regulated WT MRD1 gene and a WT MRD1 gene or a PGAL1 regulated WT MRD1 and a 5′-ins mutant MRD1 gene, were grown in galactose containing medium and pipetted onto agar plates containing galactose followed by incubation at the indicated temperature. (C) Recovery of cell growth after Mrd1 depletion. Linker 2 mutant strains and a PGAL1-MRD1 strain, were depleted of WT Mrd1 by overnight growth in glucose containing medium. Synthesis of WT Mrd1 was resumed by pipetting dilution series (10 times dilution at each step) onto agar plates containing galactose followed by incubation at the indicated temperature. The wild type strain PLY094 served as a control for cell growth. (PDF) [file pone.0175506.s001.pdf]

Linker 2 of the eukaryotic pre-ribosomal processing factor Mrd1p is an essential interdomain functionally coupled to upstream RNA Binding Domain 2 (RBD2)

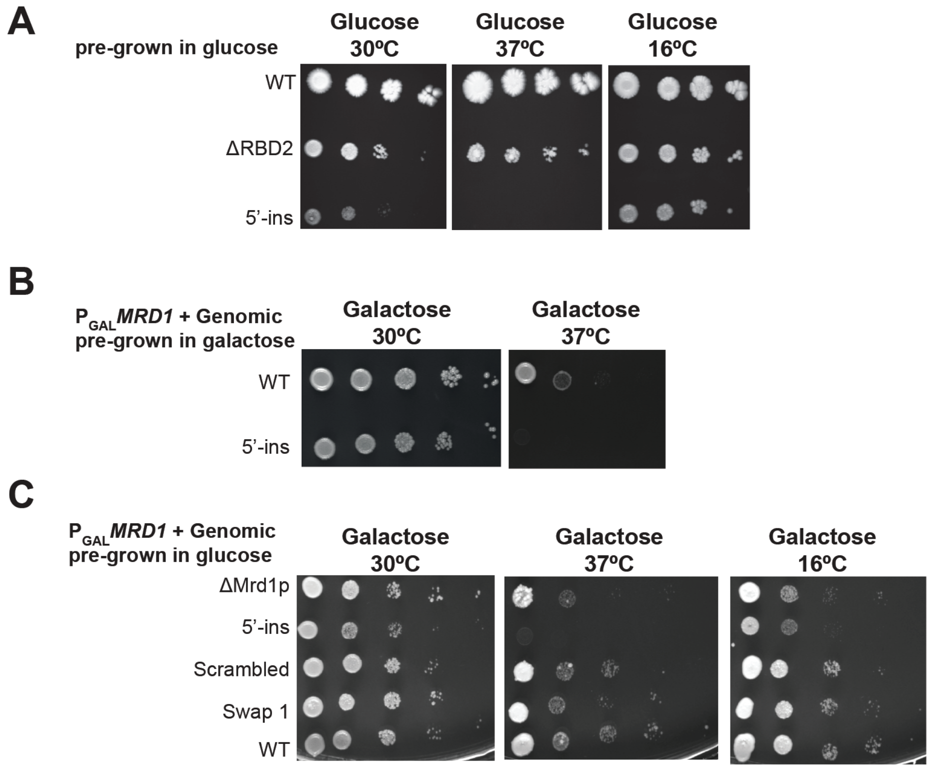

Fig. S1

(A) Growth characteristics of the ΔRBD2 and the 5′-ins mutants. WT cells and cells having mutant Mrd1 proteins, either lacking RBD2 (ΔRBD2) or having an insertion upstream of Linker 2 (5′-ins), were grown over night in glucose containing medium at 30°C and dilutions series (10 times dilution at each step) were pipetted onto agar plates containing glucose followed by incubation at the indicated temperature.

(B) Growth characteristics of cells expressing both the WT Mrd1 and the 5′-ins mutant Mrd1 proteins. Cells containing either a P_{GAL1} regulated WT *MRD1* gene and a WT *MRD1* gene or a P_{GAL1} regulated WT *MRD1* and a 5′-ins mutant *MRD1* gene, were grown in galactose containing medium and pipetted onto agar plates containing galactose followed by incubation at the indicated temperature.

(C) Recovery of cell growth after Mrd1 depletion. Linker 2 mutant strains and a P_{GAL1}-*MRD1* strain, were depleted of WT Mrd1 by overnight growth in glucose containing medium. Synthesis of WT Mrd1 was resumed by pipetting dilution series (10 times dilution at each step) onto agar plates containing galactose followed by incubation at the indicated temperature. The wild type strain PLY094 served as a control for cell growth.
