# Supplementary material for: Linker 2 of the eukaryotic pre-ribosomal processing factor Mrd1p is an essential interdomain functionally coupled to upstream RNA Binding Domain 2 (RBD2)
Source: PLoS One. 2017 Apr 7;12(4):e0175506. doi: 10.1371/journal.pone.0175506 (PMC5384785; doi:10.1371/journal.pone.0175506)
Supplement: S2 Fig — (A) Reduced levels of 18S rRNA in the Linker 2 mutants that display growth defects. Quantification of the gel from Fig 3. (B) 20S pre-rRNA levels are drastically reduced in all Linker 2 mutants that display growth defects. Quantification of the gel from Fig 3. (C) 35S pre-rRNA is accumulated in the mutants that display growth defects. Quantification of the gel from Fig 3. (D) Additional quantification of 18S versus 25S for the strains with genomic MRD1 mutant genes. Since the EtBr image in Fig 3, generated by Gel Doc™ Ez Image System (BioRad) contained overexposed pixels and also to verify the method to measure band intensity used, we ran another quantification experiment, which showed essentially the same results. Columns show quantification of 18S/25S ratios with error bars showing the average deviation of double samples. (PDF) [file pone.0175506.s002.pdf]

Linker 2 of the eukaryotic pre-ribosomal processing factor Mrd1p is an essential interdomain functionally coupled to upstream RNA Binding Domain 2 (RBD2)

A

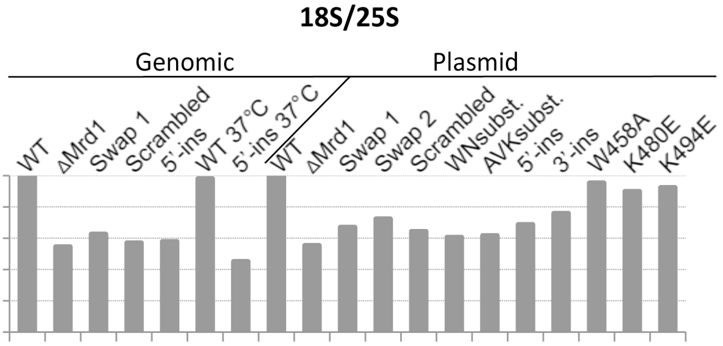

B

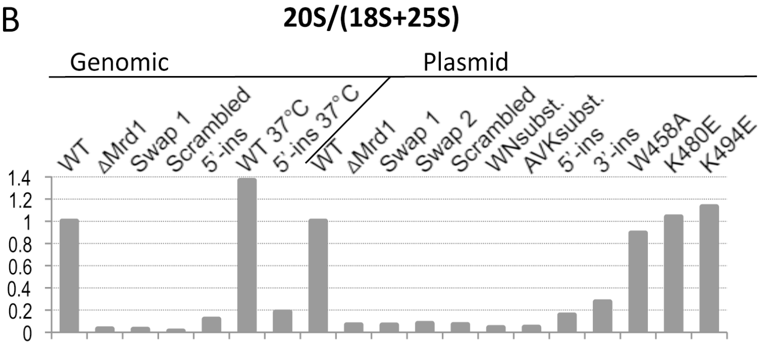

C

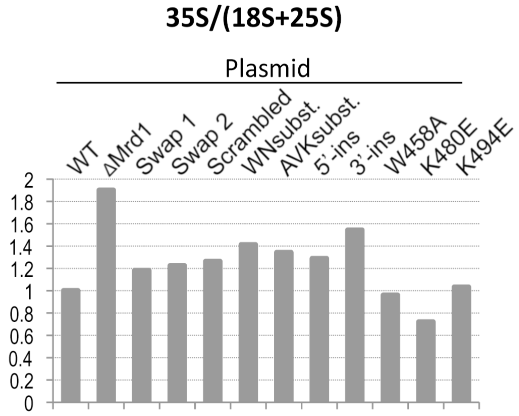

D

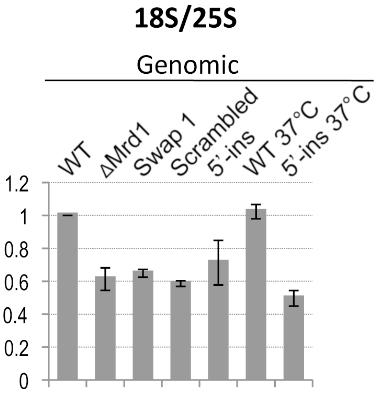

Fig. S2

(A) Reduced levels of 18S rRNA in the Linker 2 mutants that display growth defects. Quantification of the gel from Fig. 3.

(B) 20S pre-rRNA levels are drastically reduced in all Linker 2 mutants that display growth defects. Quantification of the gel from Fig. 3.

(C) 35S pre-rRNA is accumulated in the mutants that display growth defects. Quantification of the gel from Fig. 3.

(D) Additional quantification of 18S versus 25S for the strains with genomic *MRD1* mutant genes. Since the EtBr image in Fig. 3, generated by Gel Doc™ Ez Image System (BioRad) contained overexposed pixels and also to verify the method to measure band intensity used, we ran another quantification experiment, which showed essentially the same results. Columns show quantification of 18S/25S ratios with error bars showing the average deviation of double samples.
