# Supplementary material for: Linker 2 of the eukaryotic pre-ribosomal processing factor Mrd1p is an essential interdomain functionally coupled to upstream RNA Binding Domain 2 (RBD2)
Source: PLoS One. 2017 Apr 7;12(4):e0175506. doi: 10.1371/journal.pone.0175506 (PMC5384785; doi:10.1371/journal.pone.0175506)
Supplement: S1 Table — (PDF) [file pone.0175506.s003.pdf]

Linker 2 of the eukaryotic pre-ribosomal processing factor Mrd1p is an essential interdomain functionally coupled to upstream RNA Binding Domain 2 (RBD2)

Table S1. Names and genotypes of strains used in this study

Strain	Genotype	Ref.
ASY041	*MATα; ura3-52; leu2-3, 112; his3Δ200; trp1Δ; lysΔ201; mrd1-ΔRBD2-HTP-TRP1*	[1]
ASY057	*MATα; ura3-52; leu2-3, 112; his3Δ200; trp1Δ; lysΔ201; GAL2; MRD1-HTP-TRP1*	[1]
CAY1045	*MATa; his3Δ1; leu2Δ0; ura3Δ0*	[2]
Ds1-2b	*MATα; trp1Δ63; his3Δ200; ura3-52; leu2Δ1*	[3]
FLY001	*MATα; ura3-52; his3Δ200; trp1Δ; lysΔ201; GAL2; MRD1-HTP-TRP1; ade2Δ::[P_{GAL1}-3HA-MRD1-LEU2]*	This study
FLY002	*MATα; ura3-52; his3Δ200; trp1Δ; lysΔ201; mrd1-ΔLinker 2::klURA3; TRP1; ade2Δ::[P_{GAL1}-3HA-MRD1-LEU2]*	This study
FLY003	*MATα; ura3-52; his3Δ200; trp1Δ; lysΔ201; GAL2; mrd1-Swap 1-HTP-TRP1; ade2Δ::[P_{GAL1}-3HA-MRD1-LEU2*	This study
FLY004	*MATα; ura3-52; his3Δ200; trp1Δ; lysΔ201; mrd1-Scrambled-HTP-TRP1; ade2Δ::[P_{GAL1}-3HA-MRD1-LEU2]*	This study
FLY008	*MATα; ura3-52; his3Δ200; trp1Δ; lysΔ201; mrd1-Swap 1-HTP-TRP1; ade2Δ::[P_{GAL1}-3HA-MRD1-LEU2]; rpa12Δ::klURA3*	This study
FLY009	*MATα; ura3-52; his3Δ200; trp1Δ; lysΔ201; mrd1-5'-ins-HTP-TRP1; ade2Δ::[P_{GAL1}-3HA-MRD1-LEU2]*	This study
FLY019	*MATα; trp1-Δ63; his3-Δ200; leu2-Δ1; rpa12Δ::klURA3*	This study
PLY094	*MATa; ura3-52; leu2-3, 112; his3Δ200; lysΔ201*	[4]
PLY178	*MATa; ura3-52; leu2-3, 112; his3Δ200; trp1Δ; lysΔ201; hisMX6-P_{GAL1}-3HA-MRD1*	[4]

References

1. Segerstolpe Å, Granneman S, Björk P, De Lima Alves F, Rappsilber J, Andersson C, et al. Multiple RNA interactions position Mrd1 at the site of the small subunit pseudoknot within the 90S pre-ribosome. Nucleic Acids Res. 2013;41: 1178–1190. doi:10.1093/nar/gks1129

2. Gowda NKC, Kaimal JM, Masser AE, Kang W, Friedlander MR, Andreasson C. Cytosolic splice isoform of Hsp70 nucleotide exchange factor Fes1 is required for the degradation of misfolded proteins in yeast. Mol Biol Cell. 2016;27: 1210–1219. doi:10.1091/mbc.E15-10-0697

3. Nissan TA, Baßler J, Petfalski E, Tollervey D, Hurt E. 60S pre-ribosome formation viewed from assembly in the nucleolus until export to the cytoplasm. EMBO J. 2002;21: 5539–5547. doi:10.1093/emboj/cdf547

4. Lundkvist P, Jupiter S, Segerstolpe A, Osheim YN, Beyer AL, Wieslander L. Mrd1p is required for release of base-paired U3 snoRNA within the preribosomal complex. Mol Cell Biol. 2009;29: 5763–74. doi:10.1128/MCB.00428-09
